# The Cyclopædia of Practical Medicine

**Published:** 1844-07

**Authors:** 


ART. V.—*The Cyclopædia of Practical Medicine.* Edited by JOHN FORBES, M.D., F.R.S., ALEX. TWEEDIE, M.D., F.R.S., and JOHN CONNOLLY, M.D. Revised, with additions, by ROBLEY DUNGLISON, M.D. Parts II., III., and IV. Philadelphia: Lea & Blanchard. 1844.

In our last number we noticed the republication of this splendid work by Lea & Blanchard. We have since received three additional parts, an examination of which has confirmed us in our first impression, that as a work of reference for the practitioner—as a *cyclopædia of practical medicine*—it is admirably adapted to the wants of the American profession. In fact, it might advantageously find a place in the library of any gentleman, who has leisure and taste for looking somewhat into the nature, causes, and cure of diseases. We copy from part IV., a portion of Dr. Dunglison's remarks on *Cholera Infantum*, not as one of the most elaborate and finished articles, for it follows the long article on Epidemic Cholera, to which it is in some degree an appendix, but because the disease is rife at this season of the year, and any suggestions in relation to it will be read at present with interest. Having stated that cholera infantum is generally regarded as peculiar to the United States, that it is not known in Paris, but is very similar to a complaint in England vulgarly called "*watery gripes*," Dr. Dunglison gives the diagnosis of the disease, and then proceeds to enumerate the causes, from which point our quotation begins:

"*Causes.*—Excessive heat is unquestionably concerned in the causation. It is, indeed, in the summer months that the disease prevails so fearfully and so fatally in our cities; yet something more than heat is required to induce it extensively; for, in the country situations it is comparatively rare. Atmospheric vitiation must be combined with atmospheric heat, and hence it prevails in the most crowded alleys of cities. In cleanly and well-ventilated districts, and in large, airy apartments, deaths from it are uncommon; so at the time when the bills of mortality indicate the greatest amount of deaths, physicians who practise extensively in the better portions of cities may see but little of it. Dr. Condie states that when it occurs in the country, it is almost exclusively in low, damp and otherwise unhealthy situations; but such has not been the result of the author's experience. Age affords a predisposition. The common idea is, that children are more liable to it in the second summer than in the first; but this does not seem to be confirmed by statistical observations. From the official reports of deaths in Philadelphia and New York, it would appear that, in the former city, of 229 deaths assigned to summer complaint, 142 occurred in children under one year of age; 75 in those from one to two years; and 12 in those from two to five years. It rarely occurs in infants under three months of age.

Under the predisposition induced by great atmospheric heat and vitiation, it is clear that improper aliment may be an exciting cause; hence, children brought up on spoon-meat suffer in greater numbers than those that are nourished at the breast.

"In like manner, dentition may contribute to its production.

"*Pathological Characters.*—The mucous membrane of the bowels generally exhibits evidences of inflammation, especially of the follicles, which by some have been regarded as the primary seat of the disease. When it has existed for any time, increased redness, in points and patches, has been observed in different parts of the stomach and intestines; but attention has not generally been paid to distinguish whether these consist in simple hyperæmia or in inflammation of the lining membrane. The follicles are very generally enlarged, inflamed, and occasionally ulcerated; the enlargement, according to Dr. Homer, often resembling a sprinkling of white sand upon the surface of the mucous membrane. Occasionally, considerable portions of the digestive tube are so much contracted as scarcely to admit a small-sized quill; at other times, portions of the mucous membrane of the stomach and intestines are greatly softened. The liver is almost always enlarged. This has likewise been regarded as the primary affection; but it is more probably secondary. The encephalon, in nearly all cases, has been found in a state of hyperæmia, and in long protracted cases serous effusions have been met with in the ventricles, and at the surface of that viscus.

"*Treatment.*—The general principles of management do not differ materially from those laid down under enteritis of the mucous coat, and diarrhœa. The cases that occur in the airy parts of the town are usually managed with facility, and carried to a fortunate issue; whilst those in the narrow alleys of the worst parts too often resist every effort of the practitioner. It is all-important to remove the little sufferer from the air that has generated the disease, to another situation. This of itself is often enough to arrest the affection; but it may be necessary to combine with it a gentle laxative—as a small dose of calomel or of rhubarb and magnesia. Should the febrile action be considerable, it may be advisable to apply a few leeches to the epigastrium, more on account of the revulsion, than of benefit to be derived from the loss of blood—covering the abdomen after the leech-bites have ceased to bleed, with an emolient poultice. In other cases, especially where there is great irritability of the stomach, stimulating cataplasms or fomentations may be applied, and in protracted cases a blister. Should the disease not yield to the above treatment, small doses of the mild chloride of mercury may be given frequently, to produce a new action. if the child be under two years of age, which, as has been seen, is usually the case. After this age, there is greater danger of inducing severe stomatitis. Should signs of acidity exist, magnesia in the early period, and prepared chalk subsequently, may be useful adjuncts. Hence, the hydrargyrum cum cretâ of the pharmacopœias is a convenient preparation. Where irritability of the stomach is present, which is not relieved by external revellents, injections of salt and water may be used in the way of revulsion; or gentle excitants may be given internally, as a drop or two of oil of aniseed in sugared water, or five or six drops of sulphuric ether in the same vehicle.

"It need scarcely be said, that should irritation from teething exist, the gums should be freely divided, and should even the tooth not be set at liberty, the revulsion, thus induced, may prove salutary. The warm bath is beneficial in all periods of the disease. In the more protracted cases, astringents become necessary. The acetate of lead has been highly extolled by many. The oil of turpentine has been advised when there is flatulent distention of the stomach and bowels; and with the same view, a few drops of any of the essential oils may be administered. Great caution must, however, be had in the use of both excitants and astringents. During the convalescence any of the milder tonic infusions may be prescribed.

"Throughout the disease, care must be taken in regard to the diet; and, where practicable, the child should be restricted to the mother's milk. Where this cannot be done, boiled milk and water, with any of the farinaceous preparations, as sago or arrowroot, may be used. By many, the infusion of benne-leaves or gum-water is given freely; but it may be questioned whether these mucilaginous substances are of much benefit; they may tend to shield and soothe the part with which they may come in contact, but they are difficult of digestion. The appetite is apt to be very capricious—a great desire being occasionally shown for salted meat, salt fish, butter, &c.; and it has been esteemed proper to gratify it occasionally to a certain extent. Doubtless, in many cases, no evil has resulted from such indulgence, but it must necessarily be hazardous. To allay the intense thirst, iced water may be allowed; or the infant may be permitted to suck ice contained in a piece of gauze.

"At all periods of the disease, one of the most important curative agents is change of air. If the child be removed into the country, this is the best course; but where this cannot be accomplished, riding or carrying the child about in the open air is of essential service.

"In the way of prevention, removal during the summer heats to the country; or, if compelled to remain in town, sleeping on a mattrass, with the door and windows of the chamber open; the daily use of the tepid or cold bath; exercise in the open air, and, if the child be weaned, well-regulated diet, with especial abstinence from unripe fruits and the succulent vegetables. The constant use of a flannel bandage, even through the heat of summer, has appeared to the writer an important prophylactic."

If we were to propose any modification in Dr. Dunglison's method of treating cholera infantum, that which we should suggest would be that calomel should have a little greater prominence in it. Our experience is, that calomel is the only remedy which can be relied on in cholera infantum to check the watery discharges, and bring about healthy alvine dejections. Calomel rarely fails to do this, if persevered in, and often accomplishes it very speedily; but, unfortunately, the causes continuing to operate, in too many cases the disease returns again and again, and is not finally subdued until cold weather sets in. In the use of ice, and ice-water, which Dr. Dunglison, in other places more highly than here, recommends in this and its kindred affections, we heartily concur. We have very often witnessed the happiest effects, and never any but happy effects, from the practice. The relief to the little sufferer, in cases of gastric distress accompanied by fever, is so prompt and striking, that no one who has witnessed it will ever forget it. We might also speak of the advantages of the tepid, or cold-bath, where fever and inordinate thirst are present, in cases of which sort we have seen this remedy exert a most favorable influence. We will only add, that we have met with cases of this disease which were clearly complicated with intermittent fever. On observing such patients carefully, distinct febrile paroxysms are perceived, during which all the symptoms are very much aggravated. In such cases the sulphate of quinine is indicated. We have given it successfully in grain doses to children 18 months old, two or three times during the morning, in anticipation of a chill. In solution, it is given to children with less difficulty than would be expected from its extreme bitterness. Blisters in infants are prone to run into mortification, of which we have seen melancholy instances. They are to be applied with extreme caution.

We close our notice with an extract from the article on constipation which we recommend to the careful study of the young practitioner:

"In the costiveness which depends upon mechanical obstruction of the fæces in their passage through the rectum, we have before observed that pains of a spasmodic nature in the course of the colon occur; and there is also often a sensation as if a cord was tightly tied around the abdomen. The great distension to which the colon is occasionally subjected from the accumulation of fæces, owing to the costiveness produced by the strictured state of the rectum, may explain these distressing feelings. The enormous extent to which the colon becomes dilated, in some instances, in consequence of this mechanical obstacle to its passing forward its contents, is truly astonishing. Its circumference, under such circumstances, has been found, throughout its whole course, equal to that of a stomach of the full size, and the muscular fibres and longitudinal bands proportionably increased in size.

"It is usually in cases where the costiveness has been in existence for a very long period that the colon is found so enormously distended, and when stricture of the rectum has proceeded gradually, and has at length arrived at so aggravated a state that scarcely any fæces can pass through the contracted part, that this dilated condition of the colon is most remarkably developed. The following case occurred some years ago at the Worcester Infirmary. The patient, Elizabeth Stockall, was for three or four years the victim of stricture in the lower part of the rectum, which was sufficiently near the sphincter ani to admit of her becoming relieved from time to time by dividing the stricture with a bistoury; and for two years the accumulated fæces arising from the costiveness were, as it became necessary, removed either by a division of the stricture, or by the use of glysters. She never complained of much pain down at the strictured part of the rectum, but very often in the direction of the sigmoid flexure of the colon. There was in this case so little contractile power in the rectum below the stricture, that the fæces passed by little and little through the contracted part, and accumulated in large quantities in a pouch below the contraction, and it was necessary to wash this accumulation away by the repeated use of clysters. At length, the strictured part would no longer admit of the passage of any fæces, and stercoraceous vomiting took place, soon after which her existence terminated. On examination of the abdominal viscera, the first thing that claimed attention was the arch of the colon distended to the size of a large stomach, and completely concealing the small intestines. On further tracing the colon, the dilatation of it was found equally great from the cæcum to its termination in the rectum. The sigmoid flexure of the colon was displaced from its usual situation, and lay across from the left to the right iliac region. The rectum also, from its commencement to the strictured part, near the anus, was equally distended. The fæcal matter contained in the cæcum was hardened, but that in the colon and rectum was soft and pultaceous." Y.

